# Gut Microbiota Was Involved in the Process of Liver Injury During Intra-Abdominal Hypertension

**DOI:** 10.3389/fphys.2021.790182

**Published:** 2021-12-10

**Authors:** Zeyu Zhao, Zhengchang Guo, Zhengliang Yin, Yue Qiu, Bo Zhou

**Affiliations:** Department of General Surgery, The First Affiliated Hospital of Anhui Medical University, Hefei, China

**Keywords:** intra-abdominal hypertension, abdominal compartment syndrome, fecal microbiota transplantation, 16S rRNA, gut microbiota dysbiosis

## Abstract

**Background:** Intestinal damage caused by intra-abdominal hypertension (IAH) and abdominal compartment syndrome (ACS) can lead to the ectopic gut microbiota, which can contribute to liver injury *via* portal veins. Therefore, it is speculated that gut microbiota disorder caused by IAH/ACS may result in liver injury. The relationship between gut microbiota and IAH/ACS-related liver injury was investigated in this study.

**Methods:** A model of IAH was established in rats, and 16S rRNA sequencing was analyzed for gut microbiota in the feces of rats. The elimination of gut microbiota was completed by antibiotics gavage, and fecal microbiota transplantation (FMT) was used to change the composition of gut microbiota in rats.

**Results:** In addition to the traditional cause of liver blood vessel compression, liver injury caused by IAH was also associated with gut microbiota dysbiosis. Gut microbiota clearance can relieve liver injury caused by IAH, while FMT from IAH-intervened rats can aggravate IAH-related liver injury.

**Conclusion:** The gut microbiota was one of the most important factors contributing to the IAH-related liver injury, and the JNK/p38 signaling pathway was activated in this process.

## Introduction

Intra-abdominal hypertension (IAH) and abdominal compartment syndrome (ACS) are increasingly recognized as established causes of potential complications in critically ill patients (De Laet et al., 2020). According to the consensus of the World Society for the Abdominal Compartment Syndrome (WSACS) in 2013, IAH was defined as an increased level of intra-abdominal pressure (IAP) reaching no less than 12 mmHg, and ACS was defined as a sustained elevation in IAP exceeding 20 mmHg and development of new-onset organ dysfunction (with or without an abdominal perfusion pressure under 60 mmHg) (Kirkpatrick et al., 2013). IAH/ACS influences the blood flow to various organs and affects all body systems, including circulatory disease, respiratory failure, liver failure, renal failure, and gastrointestinal dysfunction (Papavramidis et al., 2011; Reintam Blaser et al., 2017; Nazer et al., 2019).

The liver function is damaged with edema, inflammation, and necrosis when the IAP keeps elevating (Lima et al., 2017). Numerous studies have explored the reason for liver injury in IAH/ACS, and they found that elevated IAP can exert a severe impact on hepatic hemodynamics, liver perfusion, and parenchymal histology (Diebel et al., 1992; Cheatham, 2009; Inal et al., 2011; Antoniou et al., 2018; Regli et al., 2019). IAH/ACS can impair several functions of the intestinal barrier, enhance intestinal permeability, and decrease the abundance and diversity of gut microbiota (Zhao et al., 2020). Previous studies have reported the association between gut microbiota and sepsis-induced liver injury (Gong et al., 2019). However, the relationship between liver function damage and gut microbiota in IAH/ACS remains to be illustrated. Hence, we aimed to investigate the role of gut microbiota in the IAH/ACS-associated liver injury in this study.

## Materials and Methods

### Experimental Animals

Sprague-Dawley male rats (*n* = 36, weight: 200 ± 20 g) of 6–8-week-old specific-pathogen-free were obtained from the Experimental Animal Center of Anhui Medical University, Hefei, China. The animals were housed in a controlled environment (25 ± 2°C, 50–60% humidity, 12 h/12 h light/dark cycle) and fed with standard rat chow and tap water *ad libitum*. One week of adaption was offered to all the animals before starting the study. The experimental protocol was reviewed and approved by the Animal Ethics Committees of Anhui Medical University (Approval number: 20170354). All efforts were taken to minimize the animal suffering during experiments.

### Intra-Abdominal Hypertension Model Established

Rats fasted for 12 h while water was free to access before operation. The IAH model was established according to the method previously described (Gong et al., 2011; Leng et al., 2016). In brief, rats were anesthetized with sodium pentobarbital 40 mg/kg by intraperitoneal injection. Their body was kept warm by a thermostatic blanket. After the abdomen was shaved and sterilized, a disposable venous infusion needle with a micro-infusion pump was punctured on the abdominal cavity for nitrogen gas insufflation. The IAP was raised exceeding 20 mmHg and maintained for 4 h. The sham group was subjected to the same operation without nitrogen gas insufflation. After 4 h of IAH, rats were sacrificed by an overdose of sodium pentobarbital (160 mg/kg). Samples of whole blood, hepatic tissue, jejunum tissue, and feces in the cecum were collected.

### Experimental Groups

Rats were randomly divided into six groups (*n* = 6 in each group): (1) Control group (sham operation, Con); (2) IAH 4-h group, a sustained elevation in IAP exceeding 20 mmHg for 4 h; (3) PBS group, in which the rats were administered PBS intragastrically once daily for 5 consecutive days; (4) ABX group, in which the rats were administered antibiotics intragastrically to deplete the gut microbiota; (5) FMT-C group, in which the rats received feces oral gavage from the control group rats; (6) FMT-I group, in which the rats received feces oral gavage from the IAH 4-h group rats.

### Gut Microbiota Clearance and Fecal Microbiota Transplantation

Antibiotics (vancomycin, 100 mg/kg; ampicillin, 200 mg/kg; metronidazole, 200 mg/kg; and neomycin sulfate, 200 mg/kg) were administered to rats intragastrically one time daily for 5 consecutive days to deplete the gut microbiota (Gong et al., 2021). Another group of rats was administered as described above, except the antibiotics were replaced by PBS. FMT in recipient rats was performed according to the several modified methods described previously (Gong et al., 2018; Xia et al., 2021). In brief, the stool contents of the donor rats (control group rats or IAH 4-h group rats) were harvested and resuspended in sterile PBS at 0.05 g/ml and centrifuged at 1,000 rpm for 15 min. The supernatants were aliquoted and frozen at −80°C until used. Recipient rats were orally gavaged with antibiotics as mentioned above for 5 consecutive days to deplete the gut microbiota, and then, an amount of 1 ml donor fecal contents were administered to the recipient rats by gavage once daily for 3 consecutive days.

### Biochemical Analysis

Blood was obtained *via* cardiac puncture before the rats were sacrificed. The blood samples were centrifuged at 3,000 × *g* for 10 min at 4°C to collect the serum. An automated analyzer (HITACHI 3100, Tokyo, Japan) was used to measure the levels of serum aspartate aminotransferase (AST) and alanine aminotransferase (ALT).

### Gut Microbiota 16S rRNA-Sequencing Analysis

Total genome DNA from collected feces was extracted and monitored by Equalbit dsDNA HS Assay Kit. The V3–V4 hypervariable regions of the 16S rDNA sequence were selected for generating amplicons and following taxonomy. A linker with an index was added to the end of the PCR product of 16S rDNA by PCR for NGS sequencing, and the library was purified with magnetic beads. The library was quantified to 10 nM, and PE250/FE300 paired-end sequencing was performed according to the Illumina MiSeq/NovaSeq (Illumina, San Diego, CA, United States) instrument manual. A quality filtered, purified, chimeric sequenced, VSEARCH clustering sequence was used for operational taxonomic unit (OTU) clustering (the standard of sequence similarity is set to 97%). Then, the ribosomal database program classifier Bayesian algorithm was used to analyze the representative sequences of OTU species taxonomy. The community composition of each sample was statistically analyzed under different species classification levels. Based on the OTU analysis results, the method of random sampling sample sequences was used; Chao1 index and Shannon alpha diversity index were calculated; community species abundance, diversity of rarefaction curves, and rank-abundance graph were drawn. Principal components analysis (PCA) was performed based on the sample OTU abundances table. Principal coordinates analysis (PCoA) and non-metric multidimensional scaling (NMDS) were calculated based on the distance between the Bray–Curtis matrix. Linear discriminant analysis effect size (LEfSe) was used to compare the hierarchy of evolution between-group differences of microbial community structure and species, which were shown by the species and the branching tree diagram. Metastats gap analysis was used to present the species abundance differences of microbial communities.

### Hematoxylin and Eosin Staining and Liver Injury Scoring

The liver tissues of rats were fixed in 10% formaldehyde, embedded in paraffin, and sectioned into 4 μm thick layers. After staining with hematoxylin and eosin (H&E), the pathological changes of images were detected using the TissueFAXSi-plus imaging system (TissueGnostics, Vienna, Austria). The histological changes of the hepatic sections were evaluated by pathologists *via* a blind test. The histological score of the liver was graded from 0 to 4 based on the severity of the inflammatory and necrosis process. The score was calculated by accumulating all the scores of each parameter and a maximum score was 12 (Ko et al., 2020).

### Immunohistochemical Staining

The expression of claudin-1, occludin, and ZO-1 in the paraffin-embedded sections was detected by immunohistochemical staining. Briefly, the sections were incubated with claudin-1 (1:200, 28674-1-AP, Proteintech, Rosemont, IL, United States), occludin (1:200, 27260-1-AP, Proteintech, Rosemont, IL, United States), and ZO-1 (1:500, ab221547, Abcam, CA, United States) antibody overnight at 4°C after receiving antigen recovery. Then, incubation with goat anti-rabbit IgM (1:200, ab2891, Abcam, CA, United States) for 1 h was carried out. Images were scanned by the TissueFAXSi-plus imaging system (TissueGnostics, Vienna, Austria).

### Western Blot

The rat hepatic tissues were extracted with RIPA lysis buffer (Beyotime, Jiangsu, China) to obtain protein samples. The concentration of protein was detected by the bicinchoninic acid assay kit (Beyotime, Jiangsu, China). Equal volumes of protein samples were fractionated by SDS-PAGE and then transferred to the PVDF membranes. Later, membranes were blocked in 5% skimmed milk for 1 h at room temperature. After incubation with primary antibody ERK1/2 (1:5,000, ab184699, Abcam, CA, United States), p-ERK 1/2 (1:1,000, ab201015, Abcam, CA, United States), JNK (1:2,000, ab208035, Abcam, CA, United States), p-JNK (1:5,000, ab76572, Abcam, CA, United States), p38 (1:2,000, 66234-1-Ig, Proteintech, Rosemont, IL, United States), p-p38 (1:2,000, 28796-1-AP, Proteintech, Rosemont, IL, United States) overnight at 4°C, HRP-conjugated secondary antibodies (1:1,000, SA00001-1/SA00001-2, Proteintech, Rosemont, IL, United States) were incubated for 1 h at room temperature. Protein bands were visualized by the BeyoECL Plus assay kit (Beyotime, Jiangsu, China). The quantification of target protein was analyzed using ImageJ software (National Institutes of Health, Bethesda, MD, United States).

### Statistical Analysis

All data were statistically analyzed by GraphPad Prism 8.4. The results were expressed as mean ± SD and evaluated using a two-tailed Student’s *t*-test. *p* < 0.05 was considered statistically significant between groups.

## Results

### Intra-Abdominal Hypertension Induced Liver Injury in Rats

**FIGURE 1:**
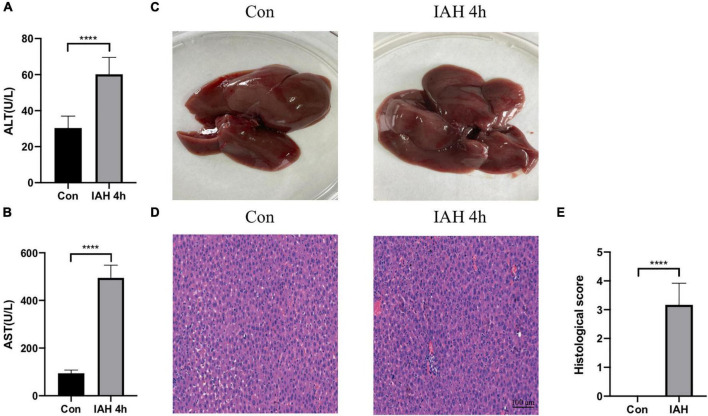
Liver injury induced by IAH. **(A,B)** The levels of plasma ALT and AST after IAH intervention for 4 h. **(C)** Representative liver morphology. **(D,E)** HE staining and the histological score of liver injury after IAH intervention for 4 h (mean ± SD. ****p < 0.0001; Con group vs. IAH 4-h group; *n* = 6 per group).

Figure 1 shows that the levels of ALT and AST in plasma were significantly increased in the IAH 4-h group (*p* < 0.05, Figures 1A,B). The appearance of the liver after H&E staining implied that rats in the IAH 4-h group also presented with more severe hepatic damage (Figures 1C–E).

### Intra-Abdominal Hypertension Induced the Gut Microbiota Dysbiosis

**FIGURE 2:**
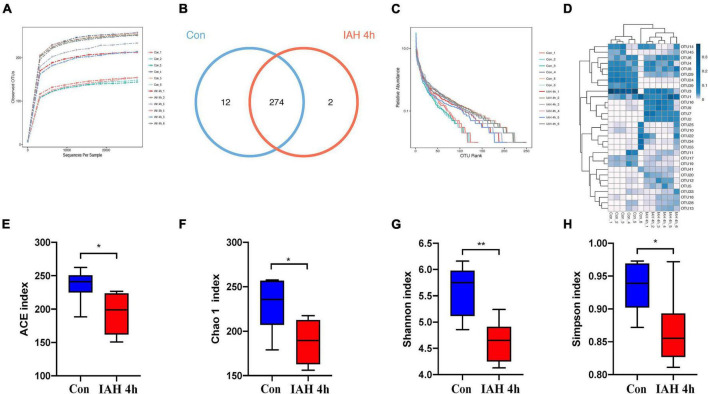
Gut microbiota dysbiosis after IAH intervention for 4 h. Rarefaction curves **(A)**, bacteria OTUs **(B)**, OTU rank curves **(C)**, heatmap **(D)**, and composition of alpha-diversity including ACE **(E)**, Chao1 **(F)**, Shannon **(G)**, and Simpson **(H)** indices between control group with IAH 4-h group (*p < 0.05; **p < 0.01; Con group vs. IAH 4-h group; *n* = 6 per group).

**FIGURE 3:**
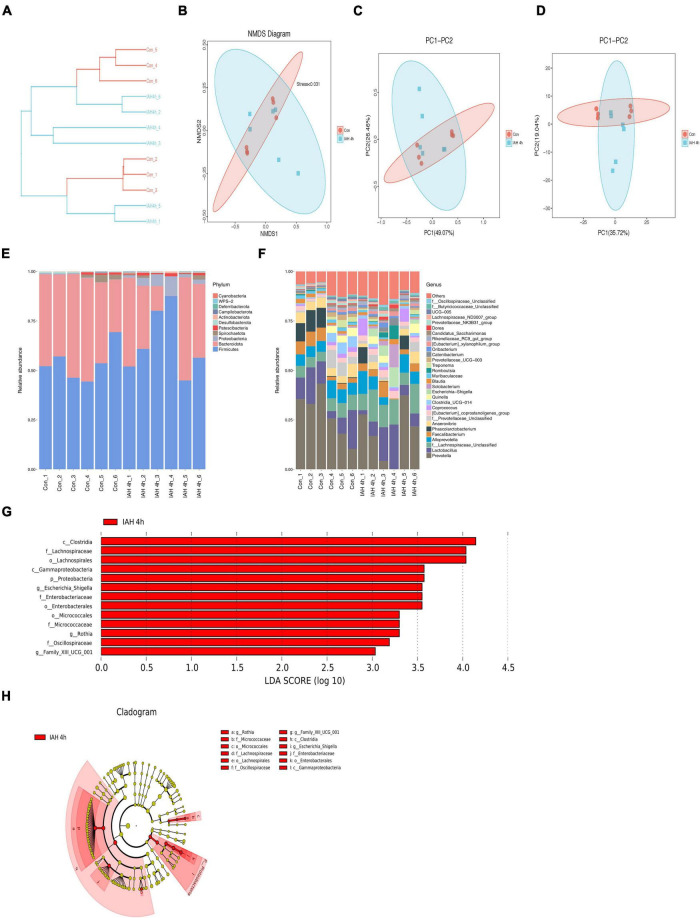
**(A)** Different branches accumulated in the IAH 4 h group and control group were, respectively, presented in the UPGMA clustering tree. The composition of beta-diversity between the control group and the IAH 4-h group. Scatter plots of NMDS **(B)**, PCoA (Bray–Curtis matrix) **(C)**, and PCA (OTU abundances) **(D)** for gut microbiota. Each symbol represents one sample. The percentage of total microbiota between the control group and the IAH 4-h group was presented at phylum **(E)** and genus **(F)** levels. The enriched bacteria in the IAH 4-h group was presented by the LEfSe analysis **(G,H)**.

An amount of 1,113,391 valid reads were acquired from 12 specimens by MiSeq sequencing (Con group and IAH 4 h group, *n* = 6 each group). The sequencing for our samples was deep enough to obtain most of the OTUs due to the rarefaction curves almost reaching the saturation plateau (Figure 2A). The Venn diagram (Figure 2B) result showed that 12 unique OTUs in the control group and 2 unique OTUs in the IAH 4-h group and 274 universal OTUs in both groups were detected. The OTUs abundance and alteration of gut microbiota in both groups were revealed by the OTU rank curve and OTU heatmap (Figures 2C,D). The alpha diversity of microbiota presented in this study illustrated that ACE (Figure 2E), Chao1 (Figure 2F), Shannon (Figure 2G), and Simpson (Figure 2H) indices were significantly lower in the IAH 4-h group than that in the control group (*p* < 0.05), indicating that the richness and diversity of gut flora in the IAH 4-h group were lower than that in the control group. The beta diversity of microbiota in the IAH 4-h group was different from the control group based on the unweighted pair-group method with arithmetic mean (UPGMA) analysis (Figure 3A), NMDS analysis (Figure 3B), PCoA (Figure 3C), and PCA (Figure 3D).

The changes in microbial composition at the class, family, genus, order, phylum, and species levels during IAH were presented in this study (Figures 3E,F and Supplementary Figure 1). At the phylum level, the most dominant phyla of the control group and the IAH 4-h group were Firmicutes, Bacteroidota, and Proteobacteria species. IAH increased the relative abundance of Firmicutes and Proteobacteria species and decreased the relative abundance of Bacteroidota species. Additionally, the relative differences in the microbial composition were further revealed by the levels of other microbial compositions. The LEfSe shows that the relative abundance of Clostridia, Lachnospiraceae, Gammaproteobacteria, Proteobacteria, Escherichia_Shigella, Enterobacteriaceae, Micrococcaceae, Rothia, Oscillospiraceae, and Family_XIII_UCG_001 was significantly higher in the IAH 4-h group (LDA score > |3|, Figures 3G,H).

### Intra-Abdominal Hypertension-Related Liver Injury Was Alleviated by ABX Pretreatment

**FIGURE 4:**
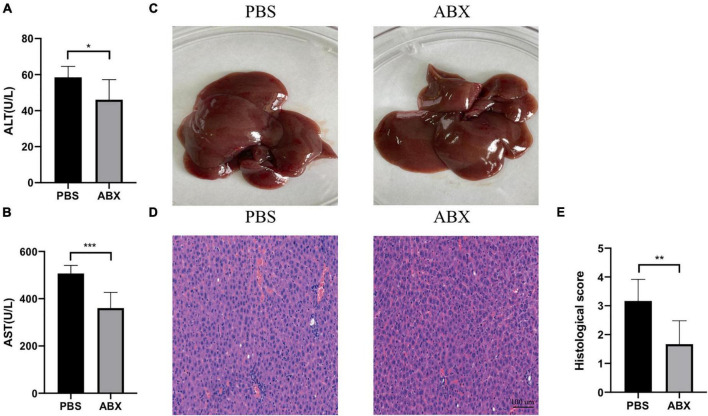
IAH-associated liver injury was alleviated by ABX pretreatment. **(A,B)** The levels of plasma ALT and AST. **(C)** Representative liver morphology. **(D,E)** HE staining and histological score of liver injury (mean ± SD. *p < 0.05; **p < 0.01; ***p < 0.001; PBS group vs. ABX group; *n* = 6 per group).

As shown in Figure 4C, the IAH-induced liver injury was significantly ameliorated in the ABX group. Compared with the PBS group, the levels of plasma ALT and AST in the ABX group were decreased (*p* < 0.05, Figures 4A,B), and the HE scores of the liver in the ABX group were also declined (*p* < 0.05, Figures 4D,E).

### Intra-Abdominal Hypertension-Related Liver Injury Was Affected by Fecal Microbiota Transplantation

**FIGURE 5:**
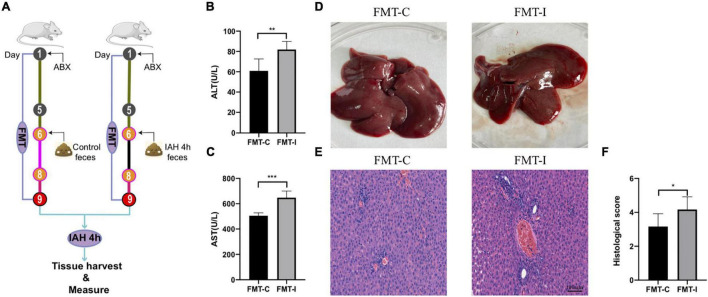
IAH-associated liver injury was aggravated by FMT of donor feces from IAH-intervened rats. **(A)** FMT operation diagram. **(B,C)** The levels of plasma ALT and AST. **(D)** Representative liver morphology. **(E,F)** HE staining and histological score of liver injury (mean ± SD. *p < 0.05; **p < 0.01; ***p < 0.001; FMT-C group vs. FMT-I group; *n* = 6 per group).

To facilitate certification of our assumption that IAH-related gut microbiota dysbiosis promotes liver damage, an FMT operation was performed. First, the rats received antibiotics mentioned above once daily for 5 consecutive days to deplete gut microbiota, and then they received donor feces resuspended in sterile PBS from control or IAH-intervened rats for 3 days (Figure 5A). The rats that received feces of the IAH-intervened group exhibited more severe hepatic injury than those that received the feces of the control group after IAH intervention (*p* < 0.05, Figures 5B–F). Hence, we summarized that gut microbiota plays a key role in IAH-related hepatic damage.

### Intra-Abdominal Hypertension Induced the Intestinal Barrier Dysfunction

**FIGURE 6:**
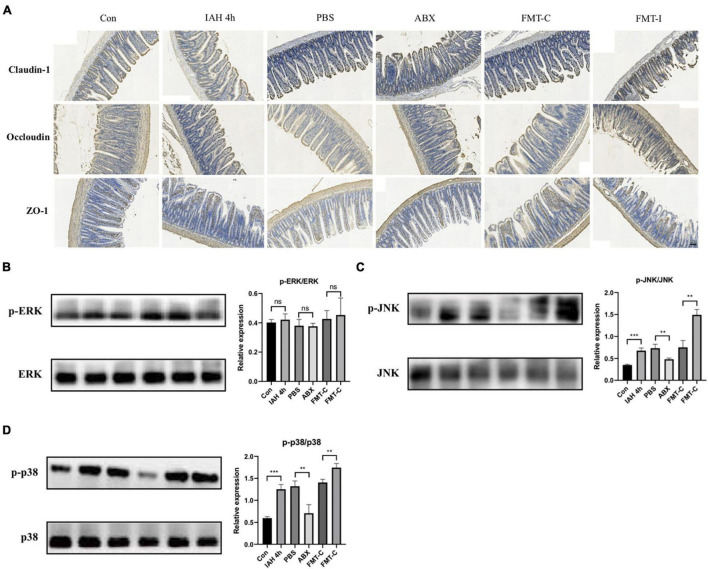
**(A)** The expression of claudin-1, occludin, and ZO-1 proteins was detected by immunohistochemistry. Protein levels of p-ERK/ERK **(B)**, p-JNK/JNK **(C)**, and p-p38/p38 **(D)** in liver tissue were measured by western blot, and quantification was performed using ImageJ software (mean ± SD. **p < 0.01; ***p < 0.001; *n* = 6 per group).

The expression levels of tight junction proteins were investigated in this study, including claudin-1, occludin, and ZO-1. The immunohistochemical staining results indicate that the expression levels of claudin-1, occludin, and ZO-1 proteins were decreased after the rats intervened by IAH for 4 h and recovered after antibiotics were administered. Furthermore, the expression levels of the three intestinal tight junction proteins were lower in the FMT-I group than that in the FMT-C group (Figure 6A).

### JNK/p38 Signaling Pathway Was Activated by the Gut Microbiota in the Intra-Abdominal Hypertension-Related Liver Injury

Figure 6 shows that the phosphorylation levels of JNK and p38 proteins were significantly higher in the IAH 4-h group than that in the control group (*p* < 0.05, Figures 6C,D), while no statistical difference was observed in the phosphorylation level of ERK1/2 protein between the two groups (Figure 6B). The phosphorylation levels of JNK and p38 proteins were lower in the ABX group compared with the PBS group and higher in the FMT-I group compared with the FMT-C group (*p* < 0.05, Figures 6C,D). The phosphorylation levels of ERK1/2 protein were also not statistically different (Figure 6B).

## Discussion

Critical illness-induced IAH/ACS may lead to hypoperfusion of both the viscera and microbiome, exerting potentially catastrophic effects on the host (Roberts et al., 2016). It seems the gut would be one of the most sensitive organs in IAH/ACS, and the gut appears to be the initial organ that causes IAH/ACS-induced multiple organ dysfunction syndromes (Cheatham, 2009). The impact of IAH/ACS on the gut is a complex multiple cascade reaction involving decreased gut perfusion, intestinal necrosis, increased permeability of bowel wall, heightened bacterial translocation, endotoxin absorption, and released proinflammatory mediators (Kirkpatrick et al., 2020). This study experimentally indicates that the elevated IAP results in the disturbance of gut microbiota homeostasis and the decreased expressions of tight junction proteins. The intestinal mucosal barrier is the first line of host defense against both encroaching enteric pathogens and invading commensal bacteria (Turner, 2009), and its dysfunction could contribute to numerous diseases, including gastrointestinal disease (Camilleri et al., 2012), liver injury (Li et al., 2021), and septic shock (Assimakopoulos et al., 2021). These demonstrate that as the first line of gastrointestinal defense, the intestinal mucosal barrier was disturbed in IAH.

In recent years, the crosstalk between the liver and gut is increasingly recognized. The relationship between the gut and liver is established by the gut–liver axis through the vascular path of the portal vein system that transfers gut-derived metabolites directly to the liver and carries bile and antibody excreted from the liver to the intestine (Albillos et al., 2020). A variety of acute and chronic liver diseases, including pyogenic liver abscess (Zheng et al., 2021), non-alcoholic fatty liver disease (Boursier et al., 2016), alcohol-associated liver disease (Gu et al., 2021), and drug-induced liver injury (Gong et al., 2018, 2021) are closely linked to disordered gut microbiota. We speculated that the gut flora may be one important switch of the host response to IAH-related liver injury due to the reason that IAH leads to gut microbiota dysbiosis, which plays a critical role in the polymicrobial sepsis-induced liver injury (Gong et al., 2019). To decipher the mechanisms, antibiotics were used to exhaust gut microbiota before IAH intervention. We found that the liver was injured by IAH intervention, and the gut microbiota depletion could reduce this effect. Furthermore, FMT of donor feces from the IAH-intervened group rats could worsen the IAH-related liver injury compared with FMT of donor feces from the control group rats.

In the process of IAH/ACS, the liver blood flow was blocked, and the liver was damaged, while the gut microbiota was disturbed in the IAH/ACS, which may contribute to an exacerbation of liver injury. Therefore, we concluded that the intestinal flora gets out of balance when IAH occurs, and the intestinal barrier was damaged and the intestinal permeability increased due to the reason that beneficial bacteria decreased and harmful bacteria enhanced, worsening the IAH-associated liver injury.

Currently, whether the Firmicutes/Bacteroidetes ratio could be a valid marker linked with metabolic alterations or not in mice and humans is controversial (Magne et al., 2020). In our work, we observed that the Firmicutes/Bacteroidetes ratio (data not shown) and the relative abundance of Proteobacteria and Escherichia_Shigella species were increased in the IAH 4 h group rats compared with the control group rats. It has been described that Proteobacteria is kept at a higher level in certain diseased host intestines compared with that in the healthy states, and many proliferating pathogens generate proinflammatory factors that lead to intestinal inflammation, increasing the level of Proteobacteria in the gut (Durban et al., 2013; Shin et al., 2015). Within the gut microbiota, Proteobacteria is the main source of lipopolysaccharides (LPS) synthesized (Lin et al., 2020). High LPS levels damage the intestinal barrier and increase intestinal permeability, resulting in the leakage of endotoxin into the plasma, triggering steatosis, inflammation, and apoptosis of the liver (Vasques-Monteiro et al., 2021). The Escherichia_Shigella could impair hepatic lipid metabolism (Liu, 2014), and the elevated levels of Proteobacteria and Escherichia_Shigella species in the IAH-intervened group rats may contribute to the IAH-related liver injury. Although the liver damage of rats in the ABX group was less severe than that in the PBS group, it was still more severe than that in the control group (Figures 1, 4). Therefore, in addition to the role of gut microbiota, there are other factors such as the compressed liver blood vessel mentioned above contributing to the IAH-related liver injury. These factors were confirmed by previous studies; however, we did not explore them in the current work.

Several studies have highlighted that the metabolites from gut microbiota could translocate into the liver through a leaky gut and then deteriorate the host inflammatory response by binding to the toll-like receptor (TLR) 4 receptors and activating the mitogen-activated protein kinase (MAPK) signaling pathway (Boulange et al., 2016). It is well known that MAPKs contain three subfamilies, ERK1/2, JNK, and p38 (Flores et al., 2019), and the phosphorylation of MAPKs was activated in the septic rat model with liver injury (Baranova et al., 2016). MAPKs are activated in hepatic ischemia-reperfusion injury, and JNK and p38 respond to stress stimulation, whereas ERK1/2 are phosphorylated by proliferative stimulation (Jimenez-Castro et al., 2019). To explore the relationship between MAPKs signaling pathway and the gut microbiota-mediated IAH-related liver injury, the protein levels of ERK1/2, JNK, and p38 were analyzed in our study. Our results illustrate that the phosphorylation of the JNK/p38 signaling pathway was activated in the process of gut microbiota-mediated IAH-related liver injury, while the ERK1/2 signaling pathway may not be activated.

## Limitations

Our study indicates that the gut microbiota has an impact on the IAH-induced liver injury, which was not reported by previous studies; however, several limitations need to be recognized. First, we did not eliminate the confounding factor that blocked portal system contributes to the IAH-induced liver injury. Second, the specific classes or metabolites of gut microbiota responsible for the IAH-induced liver injury were not investigated in this study. Further research is required to reveal the detailed mechanism of the gut microbiota associated with IAH-induced liver injury.

## Conclusion

Our results indicate that the IAH could induce gut microbiota dysbiosis, intestinal barrier dysfunction, and liver injury. Our study suggests that the disordered gut microbiota may be one of the most important regulators of IAH-induced liver injury. These results would provide novel insights for finding a new treatment target for IAH-related liver injury.

## Data Availability Statement

The raw data supporting the conclusions of this article will be made available by the authors, without undue reservation.

## Ethics Statement

The animal study was reviewed and approved by the Animal Ethics Committees of Anhui Medical University.

## Author Contributions

BZ conceived the study idea. ZZ and ZG designed the study and completed the experiment. ZY collected the data. ZY and YQ analyzed the data and drew the figures. ZZ wrote the initial draft with all other authors providing critical feedback and edits to subsequent revisions. All authors approved the final draft of the manuscript and accountable for all aspects of the work in ensuring that questions related to the accuracy or integrity of any part of the work are appropriately investigated and resolved.

## Conflict of Interest

The authors declare that the research was conducted in the absence of any commercial or financial relationships that could be construed as a potential conflict of interest.

## Publisher’s Note

All claims expressed in this article are solely those of the authors and do not necessarily represent those of their affiliated organizations, or those of the publisher, the editors and the reviewers. Any product that may be evaluated in this article, or claim that may be made by its manufacturer, is not guaranteed or endorsed by the publisher.

## Supplementary Material

Supplementary Figure 1 The percentage of total microbiota between the control group and the IAH 4-h group presented at class **(A,B)**, family **(C,D)**, genus **(E)**, order **(F,G)**, phylum **(H)**, and species levels **(I,J)**.

## Abbreviations

IAH: intra-abdominal hypertension
ACS: abdominal compartment syndrome
MOF: multiple organ failure
IAP: intra-abdominal pressure
FMT: fecal microbiota transplantation
AST: aspartate aminotransferase
ALT: alanine aminotransferase
OTU: operational taxonomic unit
PCA: principal components analysis
PCoA: principal coordinates analysis
NMDS: non-metric multidimensional scaling
LEfSe: Linear discriminant analysis effect size
H&E: hematoxylin and eosin
SD: standard deviation
UPGMA: unweighted pair-group method with arithmetic mean
MAPK: mitogen-activated protein kinase
TLR: toll-like receptor.
