# Supplementary figures and images for: Gut Microbiota Was Involved in the Process of Liver Injury During Intra-Abdominal Hypertension
Source: Front Physiol. 2021 Dec 10;12:790182. doi: 10.3389/fphys.2021.790182 (PMC8703017; doi:10.3389/fphys.2021.790182)

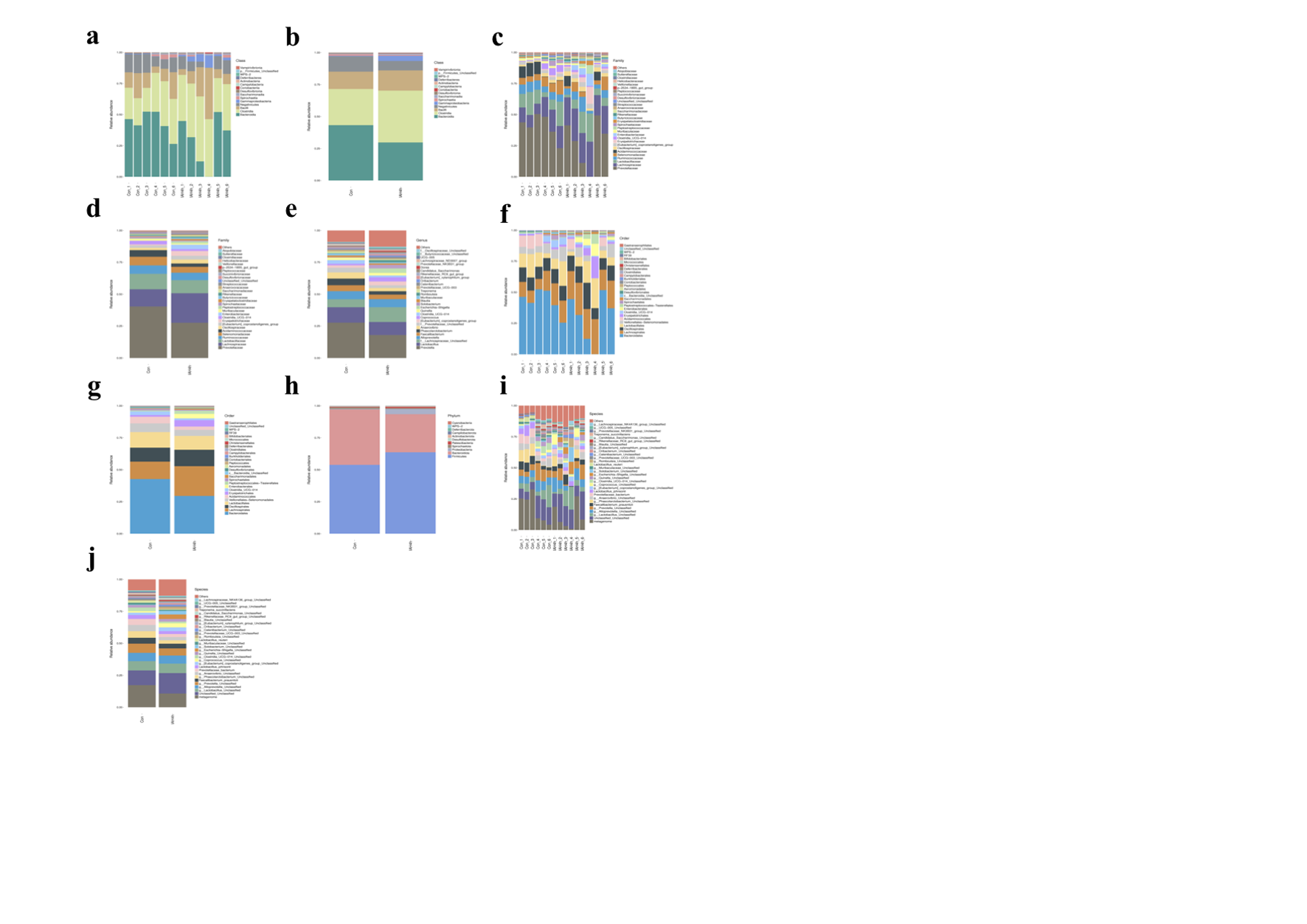

Supplement: Supplementary Figure 1 — The percentage of total microbiota between the control group and the IAH 4-h group presented at class (A,B), family (C,D), genus (E), order (F,G), phylum (H), and species levels (I,J). [file Image_1.TIFF]
